# Evolutionary History and Ongoing Transmission of Phylogenetic Sublineages of *Mycobacterium tuberculosis* Beijing Genotype in China

**DOI:** 10.1038/srep34353

**Published:** 2016-09-29

**Authors:** Qing-qin Yin, Hai-can Liu, Wei-wei Jiao, Qin-jing Li, Rui Han, Jian-ling Tian, Zhi-guang Liu, Xiu-qin Zhao, Ying-jia Li, Kang-lin Wan, A-dong Shen, Igor Mokrousov

**Affiliations:** 1 Key Laboratory of Major Diseases in Children, Ministry of Education, National Key Discipline of Pediatrics (Capital Medical University), National Clinical Research Center for Respiratory Diseases, Beijing Key Laboratory of Pediatric Respiratory Infection Diseases, Beijing Pediatric Research Institute, Beijing Children’s Hospital, Capital Medical University, Beijing, China; 2 State Key Laboratory of Infectious Diseases Prevention and Control, Collaborative Innovation Center for Diagnosis and Treatment of Infectious Diseases, National Institute for Communicable Disease Control and Prevention, Chinese Center for Disease Control and Prevention, Beijing 102206, China; 3 Laboratory of Molecular Microbiology, St. Petersburg Pasteur Institute, St. Petersburg 197101 Russia

## Abstract

*Mycobacterium tuberculosis* Beijing genotype originated in China and has undergone a dramatic population growth and global spread in the last century. Here, a collection of *M. tuberculosis* Beijing family isolates from different provinces across all China was genotyped by high-resolution (24-MIRU-VNTR) and low-resolution, high-rank (modern and ancient sublineages) markers. The molecular profiles and global and local phylogenies were compared to the strain phenotype and patient data. The phylogeographic patterns observed in the studied collection demonstrate that large-scale (but not middle/small-scale) distance remains one of the decisive factors of the genetic divergence of *M. tuberculosis* populations. Analysis of diversity and network topology of the local collections appears to corroborate a recent intriguing hypothesis about Beijing genotype originating in South China. Placing our results within the Eurasian context suggested that important Russian B0/W148 and Asian/Russian A0/94-32 epidemic clones of the Beijing genotype could trace their origins to the northeastern and northwestern regions of China, respectively. The higher clustering of the modern isolates in children and lack of increased MDR rate in any sublineage suggest that not association with drug resistance but other (e.g., speculatively, virulence-related) properties underlie an enhanced dissemination of the evolutionarily recent, modern sublineage of the Beijing genotype in China.

The advent of next-generation sequencing technologies for whole genome analysis of bacterial pathogens opened a new perspective to the more robust and more meaningful reconstructions. Nonetheless, a new technology is not a magic wand in itself since bioinformatics tools and algorithms do not always permit to achieve an unambiguous interpretation^1^. In the field of molecular evolution and phylogenetics of *Mycobacterium tuberculosis*, a consensus view on many issues is yet to be reached. Perhaps, the most controversial topics with contrasting opinions concern the location and dating of the origin of *M. tuberculosis* species and its lineages and reasons underlying the evolutionary success of certain strains^2^,^3^,^4^.

*M. tuberculosis* is a clonal species and its different lineages are marked with clearly different “*curricula vitae”*, some having declined even in the areas of their origin (*M. africanum* in West Africa being the most remarkable example), others having undergone a dramatic increase in effective population size and global dispersal. The latter may be exemplified by the Beijing family and its particular sublineages and clonal clusters.

The Beijing genotype makes up a major part of the East-Asian phylogenetic lineage of *M. tuberculosis*^5^. Two large sequence polymorphisms RD207 and RD105, are their robust markers. Based on other evolutionary markers (*mutT2* and *mutT4* genes, NTF locus), the Beijing genotype is divided into ancient (or ancestral) and modern sublineages^6^,^7^,^8^,^9^. The ancient Beijing strains with intact NTF region and wild type allele of *mutT2*-58, may be additionally differentiated by presence/absence of the RD181 region and SNP in *mutT4*-48^10^,^11^,^12^. Finally, large deletion markers permit to distinguish proto-Beijing strains that have deletion RD105 and intact RD207. This evolutionary scenario is briefly summarized in Fig. 1.

By virtue of its highest prevalence rate (~90%) in North China, this region was initially suggested and was long believed to be the area of origin of the Beijing genotype^7^,^13^. This hypothesis was recently reiterated by Merker *et al*.^3^ but challenged by Luo *et al*.^4^ who found the so called proto-Beijing strains only in the South of China. Under their non-trivial hypothesis, the origin and primary spread of the Beijing genotype were allopatric and consecutively occurred in the South and North of China, respectively^4^.

In addition to large sublineages, clonal clusters within the Beijing genotype have been identified by high-resolution genotyping (IS*6110*-RFLP and/or 24-VNTR typing). These include strain W that caused the MDR-TB outbreak in New York city in the mid-1990s^14^, Gran Canaria Beijing strain GC1237 whose transmission is ongoing^15^, and two large clonal clusters circulating in the countries of the former USSR and named 94–32 and B0/W148. The former is also termed as Russian/Asian clone CC1 defined by 24-MIRU-VNTR clustering^3^ and corresponds to the IS*6110*-RFLP-defined A0-cluster^16^,^17^. The latter (and more notorious), B0/W148 was previously defined a successful Russian clone of *M. tuberculosis*^18^. Initially, it was designated B0 (St. Petersburg Pasteur Institute database^19^) and W148 (PHRI database, NJ USA^14^) based on IS*6110*-RFLP. Based on 24-MIRU-VNTR typing, this cluster greatly overlaps with type #100–32 (MIRU-VNTRplus.org). B0/W148 is strongly associated with MDR, marked with a population growth 10-fold faster than other Beijing strains, epidemically spread across Russia (but not former Soviet Central Asia) and likely originated in Siberia^18^. Recent studies independently “rediscovered” these strains and named them East-European sublineage^20^, Resistant European cluster^21^, East European cluster 2^4^, and CC2 clonal complex^3^.

Although the Beijing genotype is justly considered to be globally widespread, the increased transmission concerns not all strains of this genetic family but only certain clusters and sublineages. To date, different proxy estimators have been used to estimate an increased transmissibility of a strain, the clustering of new cases being the most known measure. A less frequent estimator is an elevated circulation of a genotype in the child population compared to adults since TB in children is considered to be caused by recent transmission rather than by reactivation of a latent strain^22^,^23^,^24^,^25^. For example, in New York City, isolates of the LAM RD-Rio sublineage were associated with elevated transmission as was concordantly demonstrated by higher clustering, more secondary cases, and more cases in children compared to other prevalent lineages^26^. However, due to difficulty to isolate strains from children, such studies are rare.

In the present study, we sought to assess factors that have shaped historical phylogeography of *M. tuberculosis* Beijing genotype in China and could be underlying the current molecular epidemiology of the circulating strains. One may note that previous valuable Chinese studies of *M. tuberculosis* epidemiology either focused on some of the provinces or used either high resolution (IS*6110*-RFLP or 24-VNTR) or slowly evolving phylogenetic markers (e.g. spoligotypes, RD and other sublineage markers)^3^,^4^. Compared to them, we additionally discriminated within the ancient Beijing group and we analyzed patterns of molecular diversity in geographic regions and provinces across the country. Molecular profiles were compared to strain and patient data, including comparison between two target groups (adult vs children) to assess transmissibility of different strains/sublineages.

## Materials and Methods

This study was approved by the Ethics Committee of Beijing Children’s Hospital (Capital Medical University) and Chinese Center for Disease Control and Prevention (CDC).

*M. tuberculosis* strains were received from the strain library established at the Chinese CDC. The strains in the CDC strain bank have been collected since 2004 prospectively, randomly and in an unbiased manner (no special survey, quality assurance or cross-sectional study) through provincial CDCs and local TB hospitals in 25 provinces, municipalities and autonomous regions across China. The clinical strains in the bank were isolated from body fluid samples (sputum, bronchoalveolar lavage fluid, cerebrospinal fluid, pleural effusion, blood, or gastric juice) of the TB inpatients with complete clinical information. New cases were defined as TB patients who had never been treated with anti-TB drugs or who had been treated for less than 1 month. Previously treated cases were defined as patients who received anti-TB treatment for 1 month or longer.

The present study collection consisted of two groups of adults and children and was created in a two-step manner, in order to meet the specific objectives. All the strains in the CDC strain bank available from patients ≤18 years old (pediatric group) were included in this study. The other group of strains from patients >18 years old (adult group) was selected using random number table and matched the pediatric group by region and isolation time. The basic information of eligible patients was also collected.

The chosen strains were recovered on Löwenstein-Jensen medium for 4 weeks at 37 °C. The drug susceptibility testing (DST) was performed by the proportion method as recommended by WHO^27^. The concentrations of different drugs in the media were as follows: INH 0.2 μg/mL, RIF 40 μg/mL, EMB 2 μg/mL, STR 4 μg/mL, OFL 2 μg/mL, KM 30 μg/mL, and AKM 1.0 μg/mL. The strain was considered resistant to specific drug when the growth rate was more than 1% compared to the control. The isolates were defined as multidrug-resistant (MDR), extensively drug resistant (XDR) and pre-XDR according to the WHO definitions^27^.

Spoligotyping was performed using commercial kits (Ocimum Biosolutions, India) following the published protocol^28^,^29^. 24-MIRU-VNTR typing^30^ was performed using PCR followed by agarose gel electrophoresis analysis; H37Rv strain was used фы quality control and 100-bp ladder (SBS Genetech, Beijing) was used as molecular weights marker.

*mutT2* and *mutT4* genes were amplified and sequenced with primers: 5′-TAAGTCCTGGCCGACGATGG and 5′-CAACTCGATGTGCCCCTT-GG-3′ (*mutT4*), and 5′-GGCCATAAACGTCGGAAACTTG and 5′-CGCGTCCAGAAA-ACCATCGTAA-3′ (*mutT2*)^31^.

RD181 region was studied by PCR with primers 5′-CACAAATCCGCCCATACCCGTC and 5′-CGAACATCAAG-ACCGCCAGCTTC; PCR fragments of 857 bp and 147 bp indicated presence and deletion, respectively, of RD181.

**Figure 1:**
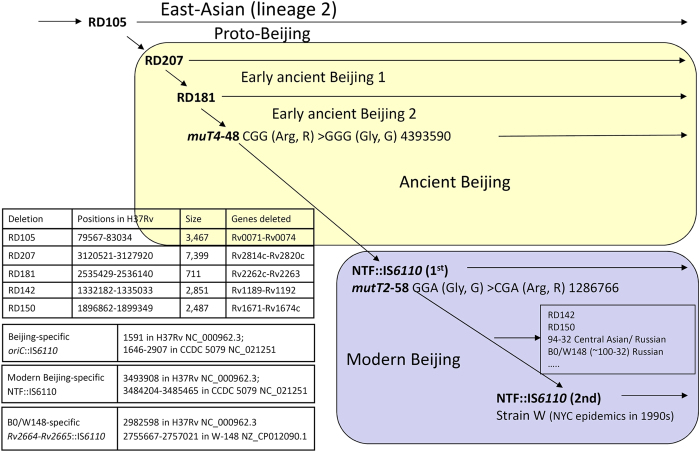
Evolutionary scenario of the *M. tuberculosis* Beijing genotype. Modified from Ribeiro *et al*.^12^ (Copyright © 2014, American Society for Microbiology) and complemented with additional information on B0/W148 strain (Mokrousov, 2013) and modern Beijing sublineage^4^.

The polymorphism of the NTF locus, i.e. presence/absence of IS*6110*, was studied as described previously^32^,^33^. Isolates with NTF::IS*6110* and/or *mutT2* 58GGA>CGA were assigned to the modern Beijing sublineage; other Beijing isolates were defined as “ancient”. In addition, “ancient” isolates with intact RD181 and/or wild type *mutT4* 48-CGG were defined as ‘early ancient” (Fig. 1).

SITVIT_WEB database (http://www.pasteur-guadeloupe.fr:8081/ SITVIT_ONLINE/index.jsp) was used to obtain SIT number for spoligoprofiles of the studied strains. MIRU-VNTRplus resource (http://www.miru-vntrplus.org/MIRU/index.faces) was used for building UPGMA (Unweighted Pair Group Method with Arithmetic Mean) tree and minimum spanning tree (MST) of 24-MIRU profiles treated as categorical variables. True clustering rate was calculated based on true clusters that included isolates of the same sublineage with identical 24-loci profiles.

The Hunter Gaston index was calculated as described^34^. A chi-square test was used to detect any significant difference between the two groups. Yates corrected χ^2^ and P-values were calculated with 95% confidence interval at http://www.medcalc.org/calc/odds_ratio.php online resource.

To achieve a more comprehensive estimation of the genetic diversity of local populations, we introduced two criteria. First, a new qualitative estimator was based on the visual assessment of the network topology that was termed as star-, chain-, or cloud-like. Second, a new quantitative measure was calculated as follows, based on VNTR typing data: CID = H/N, where CID is a cumulative index of diversity, H is mean per locus diversity and N is the level of divergence observed from the MST topology. N was calculated as ratio of number of SDLV (single/double locus variation) branches to number of other branches. N ≫ 1 means mainly recent evolution of strains in a given area and may be observed in both star-like and chain-like networks (if the latter consists of short edges).

## Results and Discussion

### Phylogeography of Beijing sublineages

The study sample included 369 isolates that represented different provinces in all geographic parts of China (Table S1). Patients were unlinked based on standard epidemiological investigation. The pediatric subgroup (n = 219) had a mean age of 12.9 ± 6.1 years and included 115 males (54.5%) and 96 females (45.5%). The adult subgroup (n = 150) had a mean age of 40.4 ± 17.6 years and included 94 males (62.7%) and 56 females 56 (37.3%). As described in the Materials and Methods section, the strains from adults were selected to match strains from children by province and year of isolation, but not by gender. This may explain a non-significant (P = 0.1) difference of the male to female ratio between adults (63:37) and children (55:45). Although beyond the scope of this study, this situation may be also due to the less prominent gender bias in TB structure in the pediatric group.

All isolates were assigned to the Beijing genotype based on spoligotyping (according to the definition of Kremer *et al*.^9^); all had the RD105 deletion. Spoligotyping revealed a relatively high diversity of the studied collection. Most of the isolates had a classical 9-signal profile but the derived variants also accounted for a certain proportion of the collection (30 derived variants, 8.1% of the 369 isolates). This diversity reflects a long history of the Beijing genotype in China, where this *M. tuberculosis* family has its origin. However, the small number of variable characters and the possibility of convergent evolution of the DR/CRISPR locus in *M. tuberculosis* prevent from making any phylogenetic inference from the spoligotyping data of the Beijing genotype. For example, spoligotype SIT190 with deleted signal #40 was found in strains of both modern and ancient sublineages.

All isolates were further subjected to the analysis of the both contrasting and complementing kind of markers: (i) large-scale sublineage markers within the Beijing family and (ii) high-resolution 24-VNTR loci. Table 1 shows a summary information on epidemiological and clinical characteristics in 3 sublineages (early ancient, ancient and modern) of the studied Beijing genotype strains. Due to PCR failure, the complete profiles were not obtained for some isolates that were therefore excluded from certain parts of the analysis. PCR failure in those strains occurred even after repeating the experiment; this situation is not unusual^35^, and may be due to specific problems of DNA composition in some samples and difficulty to amplify repeat genome regions such as, minisatellites^30^.

### Geographic distribution

**Figure 2:**
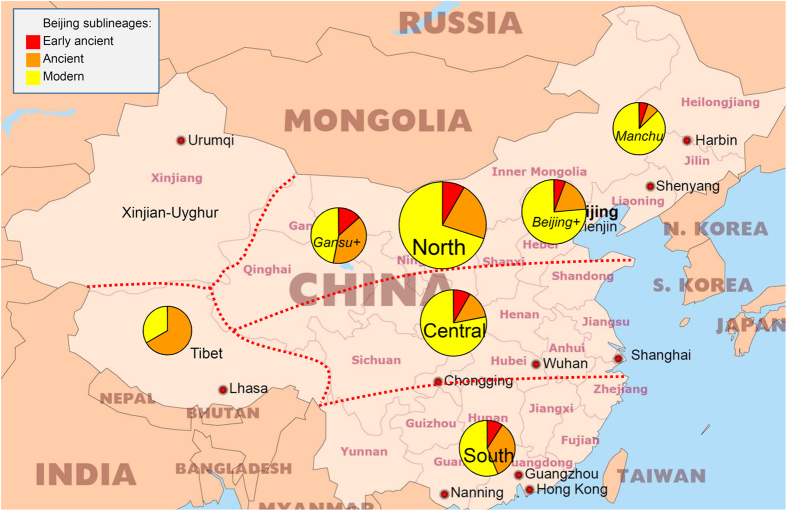
Geographic distribution of the Beijing family sublineages in five geographical regions in mainland China. Circle size is proportional to the number of isolates. Beijing+ and Gansu+ mean Beijing, Gansu and their neighboring provinces. Free map of China: http://www.freeworldmaps.net/asia/china/pdf/china_pol.pdf.

Distribution of the three sublineages of the Beijing family in China is shown in Fig. 2. We adopted the five-party subdivision into North, Center, South, Tibet, and Xinjiang-Uyghur, based on (hydro)geography and agriculture in the light of different ethnocultural and historical backgrounds. In addition, a geographical and historical Manchuria subregion was delineated within the largest North China region and includes northeastern provinces targeted in this study. Due to small sample size, the pie chart of Xinjiang-Uyghur region is not shown on the map but these strains were kept for further analysis.

Overall, some striking gradients between and within regions may be observed (Fig. 2). There is a decreasing North-to-South prevalence rate of the modern Beijing sublineage. Within northern China several gradients can be seen: (i) decreasing rate of early ancient Beijing from West to East, reaching the lowest rate in Manchuria and coinciding with a small rate of ancient Beijing and a high rate of modern Beijing); (ii) decreasing rate of modern Beijing and co-increasing rate of ancient Beijing towards Gansu and Tibet.

The ancient sublineage of the Beijing genotype is prevalent in Tibet whereas early ancient Beijing strains are absent; a possible explanation might be that there was very little exchange between Tibet and neighboring regions during the very early stages of the Beijing genotype evolution. This corroborates with the early history of Tibet being devoid of any significant Han Chinese human influx.

A comparison of our results with those previously published shows a good correlation. Our results on South China are well in line with a recent NGS study of Luo *et al*.^4^ who also showed presence of the early ancient Beijing strains in the South of China, and dissemination of modern Beijing strains out of North China (although they focused on other northern provinces). The lack of early ancient Beijing strains and the 2 to 1 proportion of ancient to modern groups, which we observe in Tibet also correlates well with the previous work^4^. Further, two provinces of historical Manchuria (Heilongjiang and Jilin) featured a very high rate of modern Beijing both in our study and in Luo *et al*.^4^.

### Beijing genotype phylogeny in China: country-wide view

The high-resolution 24-VNTR loci scheme was applied to the entire collection and the complete profile of all loci was obtained for 302 isolates with information on their sublineage status (early ancient, ancient, modern). The profiles were linked in the minimum spanning network (see Supplementary Figures S1–S4, where regions, sublineages, drug resistance and adult/child status are highlighted separately). Under further analysis, we hypothesized that core genotypes represent earlier variants in the course of the Beijing genotype evolution, while terminal types represent secondary, evolutionarily young cases, either imported or evolved *in situ*. However, these links between types should take into consideration the sublineage status: an isolate of the ancient sublineage cannot be derived from an isolate of the modern sublineage even if located more distally in MST.

Information on strain origin (province and region) permitted us to estimate the role of geographic distance in shaping mycobacterial diversity and divergence. As noted by LaPolla^36^, “all Chinese governments encouraged migration since Yin dynasty (~1600–1000 BC) up to the present. These massive movements resulted in major shifts in the overall demographics”. In the dendrogram, most of the adjacent nodes represent the same or neighboring geographic regions (Fig. S1): the total number of branches was 216, and most of them (88%, 190/216) connect types from the same or neighboring regions. This relatedness of isolates from the neighboring areas implies that people/strains tend to migrate on short- and middle-distances, rather than on large-scale ones. Accordingly, we interpret this as a confirmation that a large-scale distance remains a major factor of genetic divergence of *M. tuberculosis* in spite of human mobility across large landmasses. Although a long-distance migration has increased in China since the 1990s^37^, it does not seem to be a decisive factor in the global dissemination of local *M. tuberculosis* strains.

A closer look at the VNTR-based phylogeography of the Beijing family in China reveals a heterogeneous composition of the largest parts in MST (Fig. S1, panels A and B) that contained isolates from all regions of the country. In particular, this is true for both core and largest types 6 and 18 (Fig. S1). Both subtrees are dominated by modern Beijing strains with rare and dispersed ancient and early ancient strains (Fig. S2). An exception is the ancient branch of types 26–28 and related orphans mostly composed of strains from North China (Fig. S2C). In fact, while MST was built on all strains, it should be interpreted separately for three sublineages. The branch of ancient types 26–28 is located terminally but, apparently, it is not derived from the preceding modern type 25. Similarly, a distal branch of early ancient Beijing (types 32–33 and derived types in Fig. S2C) cannot be secondary with regard to the preceding types of the ancient Beijing sublineage.

In contrast to the densest parts, the lower part of the MST (Figs S1/S2C) consists exclusively of ancient and early ancient Beijing isolates and its core components tend to be dominated by those from Tibet (Figs S1 and S2). In this view, it seems that core type 29 is a major ancient Beijing subtype in Tibet.

We additionally looked at isolates from 4 provinces making part of the former historical Manchuria land (Jilin, Heilongjiang, Liaoning, and Inner Mongolia). However, most of the isolates are scattered across the upper and – especially - central subtrees, and no Manchu-specific types located in the core positions could be found (Fig. S1).

By definition and phylogenetic reconstruction, the Beijing genotype originated in China, and had had its long evolutionary history in this country. As a result, different Beijing subtypes have been disseminated worldwide following different flows of human migration. Some of them have become epidemic and widespread in their new countries. Geographically, the nearest to China are two Eurasian epidemic strains defined by 24-MIRU-VNTR loci: Russian type 100–32 and Asian/Russian type 94–32. Their profiles differ in loci MIRU26 and QUB26 (7 and 7 repeats for 100–32 and 5 and 8 repeats for 94–32^17^). When these profiles were placed within the Chinese tree of the Beijing genotype, they both were linked to the central and ‘modern’ part of the network (Fig. S2B).

Russian type 100–32 (B0/W148) is a major driving force of the Russian TB epidemics. It is MDR-TB associated, widespread across Russia and is isolated from Russian immigrants in Europe and the USA^18^. Its 24-MIRU-VNTR profile is SLV derived from the Shanxi and Jilin strains in this study (Fig. S2B). Both these provinces represent northeastern part of China that had historical links with the Russian Empire/USSR. Human interaction via the Chinese Eastern Railway in the 1920s was proposed as a major vehicle of penetration of the Beijing genotype strains to Russia^38^: however this scenario appears more plausible not for the entire Beijing genotype but for its B0/W148 clone.

Asian/Russian type Beijing 94–32 is highly prevalent in the former Soviet Central Asia^39^,^40^,^41^,^42^ and is one of the two largest and significant Beijing subtypes in Russia^4^,^18^,^38^. Indeed it is SLV derived from the strain from Qinghai, a neighbor province of Xinjiang-Uyghur, and of Central Asia as a whole (Fig. S2).

It may be noted that in the NGS-based tree of the Beijing family^4^, these Russian clones (named East European 1 and 2) were located distantly from Chinese isolates. In this sense, results of our study, even though based on more classical VNTR markers, suggest a link between major Russian Beijing clones and their hypothetical Chinese ancestors, although this hypothesis should be further tested on both extant and archival samples (as is the case for all in silico predictions).

### Local phylogenies and place of origin of the Beijing genotype

In view of some difference in the sublineage distribution between large geographical regions (Fig. 2), we further compared fine-tuned structures of the geographically distinct local populations across the country. To this end, we further built separate MST for six provinces, estimated their topology, and calculated a measure of the genetic diversity. Beijing capital city was not considered because of its very complex history and various migrations that likely diffused past traces of diversity^36^.

We presume that a star-like network and low diversity likely represent a recent dissemination of the genotype in an area. In contrast, a higher level of diversity along with chain-like (or especially cloud-like) topology of a network may imply a longer evolutionary history. Finally, to take into account both per locus diversity and starness of the network, we calculated a cumulated index of diversity for each targeted province as a direct proportional measure of diversity (Table S2).

**Figure 3:**
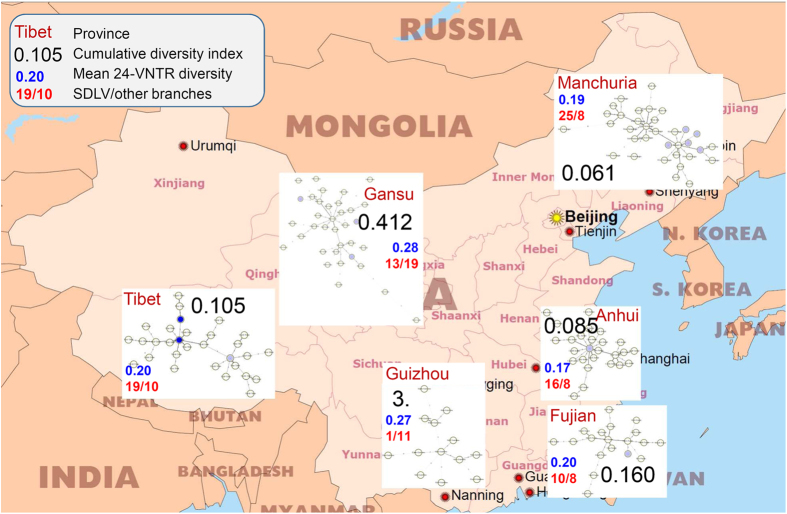
Local populations of the Beijing family in the selected regions/provinces: phylogeny, cumulative index of diversity, and other measures of diversity. Free map of China: http://www.freeworldmaps.net/asia/china/pdf/china_pol.pdf.

Extreme cases of diversity are represented by Guizhou and Anhui provinces (Fig. 3). Anhui has the lowest diversity, higher rate of SDLV and clearly star-like tree hence one may hypothesize an evolutionarily recent introduction of the Beijing genotype to this province. However, this is unexpected due to its location in the central China. In contrast, the Beijing genotype MST of Guizhou has a cloud-like topology whose long branches present triple and more locus variation. We take this situation as a reflection of the longest evolutionary history and thus an ancestral position of this province (i.e. south/southwestern China) in the evolution of the Beijing genotype.

**Figure 4:**
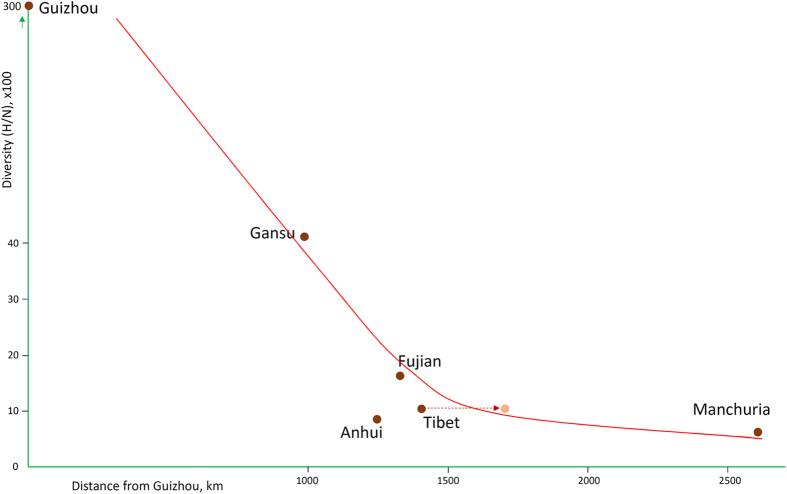
Cumulative index of diversity of Beijing genotype populations plotted against distance from Guizhou (~South China, a hypothesized place of origin of the Beijing genotype). An arrow shows a corrected position of Tibet due to mountain barriers of human migration. Exact values are shown in Supporting Table S2.

Thus, we hypothesized that Guizhou (~South China) is the place of origin of the Beijing genotype and plotted the cumulative index of diversity for each province against the distance from Guizhou (Fig. 4). A rule of decreasing diversity with increasing distance from the place of origin is well known and was proven true for different species^3^,^43^. Indeed, on the graph, the Beijing genotype diversity is decreasing with increasing distance from Guizhou (Fig. 4). This seems to confirm the recent hypothesis^4^,^44^ that the Beijing genotype originated in the South China. One should keep in mind that a number of physical barriers restrict dispersal, including mountains, large bodies of water and geographical distance. For this reason, the effective distance from Guizhou may be more than the simple geographical distance in the case of Tibet.

One may note that the ancestral position of Guizhou would explain the lowest diversity, and thus a recent introduction, of the Beijing genotype into the most distant Manchuria (high rate of SDLV branches along with low diversity of loci).

### Epidemiological findings: Adults vs children

In the subsequent analysis, we compared two age groups and contrasted pooled ancient group (ancient and early ancient strains together) against modern group of the Beijing genotype. Analysis of the complete collection of 369 isolates revealed a slight increase of the modern sublineage in the more vulnerable pediatric group but not significantly so (P = 0.14).

The 24-VNTR dendrograms were built separately for two age groups (Supporting Figs S5 and S6) and served to calculate the clustering rate which was higher in children (30.2%) compared to adults (19.6%; P = 0.05). On the MST, the isolates from the two age groups are distributed quite evenly without predominance of adults or children in any part of the network (Fig. S3). This implies that no particular clonal complex is specifically responsible for disease development in either age group.

As a next step, the clustering rate was estimated separately in the two sublineages in both age groups. In children, a significantly higher clustering was observed in modern (44/118, 37.3%) versus pooled ancient (9/64, 14.1%) isolates, P = 0.002. In adults, clustering was also higher in modern (16/79, 20.3%) versus pooled ancient (6/48, 12.5%) isolates, but not significantly so (P = 0.3). In other words, in the modern group, isolates from children clustered significantly higher (44/118, 37.3%) than isolates from adults (16/79, 20.3%), P = 0.01. In contrast, in the ancient group, isolates from children and adults showed a similar clustering rate (14.1% vs 12.5%).

Children present a special risk group for TB and are more susceptible to infection and disease by more transmissible strains^45^,^46^. However, comparative studies of adult and pediatric groups are rare due to the difficulty to isolate *M. tuberculosis* strains from children^26^,^47^,^48^. In this study, the availability of a large pediatric TB collection helped to assess transmissibility (and epidemic danger) of the particular subgroups within the Beijing genotype. Our findings suggest that strains of the modern Beijing sublineage appear to be the major driver of the TB transmission in China.

### Epidemiological findings: Drug resistance

The strains were subdivided into four major groups based on their drug resistance profile: (1) XDR and pre-XDR; (2) MDR and polyresistant; (3) monoresistant; (4) susceptible. Under certain comparisons described below, the groups (1) and (2) and groups (3) and (4) were merged.

Drug resistance and treatment status information were jointly available for 351 patients. The MDR rate was found higher in previously treated patients (59/120, 49.2%) than in newly diagnosed patients (36/232, 15.5%) (P < 0.0001, OR 5.35, 95% CI: 3.23 to 8.87). This observation is similar to the findings in the previous study carried out in China by Yang *et al*.^49^ thus confirming that MDR rate is affected by the treatment history.

An estimation of the MDR rate in ancient, early ancient and modern isolates did not reveal a difference between the three groups (Table 2). No difference between pooled ancient and modern sublineages was found, either (P = 0.5). This finding corroborates the previous observations that superspreading strains in China are not necessarily those associated with drug resistance^49^,^50^.

The information on drug resistance is highlighted in the 24-VNTR MST of all tested isolates (Fig. S4). On a whole, at a first glance, there appears a lack of particular bias of any clonal complex towards any kind of resistance/susceptibility pattern. Details on prevalence of drug resistance across clonal complexes (CC) are shown in Table 3. Overall, there was no strict correlation between resistance and sublineage/clusters. In both ancient and modern groups there are CC with a relatively increased (up to 70%) rate of the MDR/XDR strains; these are ancient CC-B and modern CC-1 groups.

Within modern sublineage, comparison of two large superculsters, CC-6 vs CC-18 did not reveal a difference in rate of poly/multidrug resistance (44% vs 49%). However, a closer look inside the large CC-6 supercluster (Fig. S4A) revealed the higher rate of poly/MDR in CC-1 vs CC-14 (65% vs 23%, P = 0.03) (Table 3). This reconfirms a different capacity of certain, relatively homogeneous clonal groups, to develop particular pathobiologically relevant properties with critical impact for strain dissemination. The drug resistance is just one example. A strong association with MDR was shown for the Russian Beijing B0/W148 strain (compared to other Russian Beijing clones) and genetically, it was due to the acquisition of particular resistance mutations^51^, and likely due to certain compensatory mutations^52^. Most recently, a similar observation was made on the 94–32-cluster and its strong link with MDR-TB in the Uzbekistan setting in Central Asia^41^.

The ancient Beijing groups on a whole were not marked with a particular link to MDR and this is in line with results by Yang *et al*.^49^. Nonetheless, a closer look at the particular CC revealed a somewhat more complex picture: all derived CC had a higher poly/MDR rate compared to the core CC-29: CCB (67%), CC-C (33%), CC-D (100%) (Table 3, Fig. S4C). Interestingly, the variation of the poly/MDR rate across mainly small ancient Beijing clusters (from 12% to 100%) was apparently higher than in mainly large modern Beijing clusters (from 23% to 65%). A longer (by definition) evolutionary history of ancient Beijing clones may be a reason for such (patho-)genetic diversity.

## Conclusions

In our opinion, this study has several points of interest, both basic and applied. We sought to correlate past evolutionary history and present molecular epidemiology of the clinically important *M. tuberculosis* Beijing genotype in its country of origin based on: (i) collection from all regions of the country (to achieve a countrywide perspective), (ii) combination of contrasting kinds of molecular markers (to achieve both an evolutionary and high-resolution view), (iii) a large collection of pediatric isolates (to answer a question of differential transmissibility of different clones and sublineages). With regard to the latter, it is worth repeating that the task of isolation of a *M. tuberculosis* strain from a child is far from trivial. In spite of certain limitations, e.g., underrepresentation of some provinces, we hope to have also made some contribution to the theoretical aspects of the phylogenetic analysis, by introducing a new estimator of population genetic diversity (cumulative index of diversity, CID) and a new terminology related to the estimation of the tree topology.

Genetic diversity within human populations decreases gradually with geographic distance from a sub-Saharan African origin, and genetic differentiation between populations also increases steadily with physical distance along landmasses^43^,^53^. More specifically in the Han Chinese, a study of genome-wide SNPs revealed: (i) a close correlation between geography and the genetic structure of the Han Chinese and (ii) a ‘North-South’ population structure although the metropolitan cities presented more diffused ‘outliers’ probably because of the impact of current migration^54^. The same pattern was previously suggested for *M. tuberculosis*^7^,^55^ and was also demonstrated in this study.

The phylogeographic patterns observed in the studied collection of *M. tuberculosis* Beijing genotype strains demonstrate that large-scale (but not middle/short-scale) distance was and remains the major factor that contributes to the genetic divergence of *M. tuberculosis* populations. Analysis of diversity and network topology of the local collections appears to corroborate a recent hypothesis about the South China origin of the Beijing genotype. Viewed within the larger Eurasian context, our results demonstrate that two epidemiologically important and fast spreading Russian (B0/W148/100-32) and Asian/Russian (A0/94-32) epidemic clones of the Beijing genotype can trace their origins to the northeastern and northwestern regions in China, respectively.

The highest clustering of the modern sublineage in children along with a lack of association of any sublineage with drug resistance suggest that not an association with drug resistance but other pathogenic (speculatively, virulence-related) properties underlie an enhanced dissemination capacity of the evolutionarily recent, modern sublineage of the Beijing genotype in China. The reason of low prevalence of strains of the ancient/ancestral Beijing sublineage may lie, hypothetically, in their reduced transmissibility.

## Additional Information

**How to cite this article**: Yin, Q.-q. *et al*. Evolutionary History and Ongoing Transmission of Phylogenetic Sublineages of *Mycobacterium tuberculosis* Beijing Genotype in China. *Sci. Rep.* **6**, 34353; doi: 10.1038/srep34353 (2016).

## Supplementary Material

Supplementary Table S1

Supplementary Table S2, Figures S1-S6

## Figures and Tables

**Table 1:** Epidemiological and clinical characteristics of patients and *M. tuberculosis* strains.

Characteristics	Early ancient (N = 33) n(%)	Ancient (N = 104) n(%)	Modern (N = 216) n(%)
Gender			
Female	15 (45.5)	42 (41.2)	90 (42.9)
Male	18 (54.5)	60 (58.8)	120 (57.1)
unknown	0	2	6
Age (years)			
≦18	20 (60.6)	54 (51.9)	134 (62.0)
>18	13 (39.4)	50 (48.1)	82 (38.0)
Treatment history			
New cases	18 (58.1)	63 (62.4)	143 (66.2)
Previously treated cases	13 (41.9)	38 (37.6)	65 (30.8)
unknown	2	3	8
Geographic distribution			
Northern	22 (66.7)	32 (31.4)	113 (54.1)
Central	6 (18.2)	18 (17.6)	50 (23.9)
Southern	5 (15.2)	20 (19.6)	28 (13.4)
X.-Uyghur	0	3 (2.9)	5 (2.4)
Tibet	0	29 (28.4)	13 (6.2)
unknown	0	2	7

Strains with available designation of either of 3 sublineages, early ancient, ancient and modern (defined by RD181, *mutT2* and *muT4* alleles [Fig. 1]).

Central: Anhui, Chongqing, Henan, Jiangsu, Shaanxi, Sichuan.

Northern: Beijing, Gansu, Hebei, Qinghai, Heilongjiang, Jilin, Liaoning, Inner Mongolia, Shandong, Shanxi, Tianjin.

Southern: Fujian, Guangxi, Guizhou, Hainan, Jiangxi.

**Table 2:** Drug resistance properties of sublineages of the Beijing genotype.

Drug-resistant type	Early ancient (N = 32) n (%)	Ancient (N = 103) n (%)	Modern (N = 214) n (%)
Sensitive	16 (50.0)	43 (41.7)	101 (47.4)
Any resistance (incl. MDR)	16 (50.0)	60 (58.3)	113 (52.8)
MDR	8 (24.2)	32 (25.6)	55 (25.6)

**Table 3:** Drug resistance in main clonal complexes (defined by combination of SLV and starness) and large single types.

Genotype, clonal complex and its position in MST (Fig. S4)	Resistant to 2 and more drugs, N and % of all in this CC	Susceptible and monoresistant, N and % of all in this CC	Total
Modern			
CC-6 supercluster	48 (44%)	62 (56%)	110
CC-6 core	28 (44%)	36 (56%)	64
*Type 6*	*2*	*5*	*7*
CC-1 distal (relative to CC-6)	11 (65%)	6 (35%)	17
*Type 1*	*2*	*3*	*5*
CC-11 distal (relative to CC-6)	6 (37%)	10 (63%)	16
CC-14 distal (relative to CC-6)	3 (23%)	10 (77%)	13
CC-18 supercluster	42 (49%)	44 (51%)	86
*Type 18*	*6*	*7*	*13*
*Type 25*	*2*	*3*	*5*
Ancient			
CC-29 core	5 (23%)	17 (77%)	22
CC-27/28 outlier	4 (33%)	8 (67%)	12
CC-A derived distal (relative to CC-29)	1 (12%)	7 (88%)	8
CC-B intermediate, derived from CC-29	4 (67%)	2 (33%)	6
CC-C derived from CC-29	2 (33%)	4 (67%)	6
CC-D derived from CC-29	5 (100%)	0	5
Early ancient			
CC-32	4 (50%)	4 (50%)	8

Number of clonal complexes (CC) is not consecutive; it is defined by the number of its central type. Data in columns are not additive since some superclusters and CC include large types.
