# Supplementary material for: Evolutionary History and Ongoing Transmission of Phylogenetic Sublineages of Mycobacterium tuberculosis Beijing Genotype in China
Source: Sci Rep. 2016 Sep 29;6:34353. doi: 10.1038/srep34353 (PMC5041183; doi:10.1038/srep34353)

Evolutionary History and Ongoing Transmission of Phylogenetic Sublineages of *Mycobacterium tuberculosis* Beijing Genotype in China

Qing-qin Yin, Hai-can Liu, Wei-wei Jiao, Qin-jing Li, Rui Han, Jian-ling Tian, Zhi-guang Liu, Xiu-qin Zhao, Ying-jia Li, Kang-lin Wan, A-dong Shen, Igor Mokrousov

Supplementary Table S2. Genetic diversity of local *M. tuberculosis* Beijing genotype populations.

Province, region	H, mean diversity of 24 loci	N, number of SDLV branches /other branches	Cumulative index of diversity, H/N	Distance from Guizhou*, km
Tibet	0.2	19/10=1.9	0.105	1405
Gansu	0.28	13/19=0.68	0.412	986
Manchuria	0.19	25/8=3.13	0.061	2649
Guizhou	0.27	1/11=0.09	3	0
Fujian	0.20	10/8=1.25	0.160	1364
Anhui	0.17	16/8=2	0.085	1189 1260

*Distance between Bijie (the largest city in Guizhou) and the largest city of the respective province/region, calculated using: http://www.distancefromto.net/

Supplementary Figure S1. Minimum spanning tree of 24-VNTR loci with highlighted regions of strain isolation.

(A)

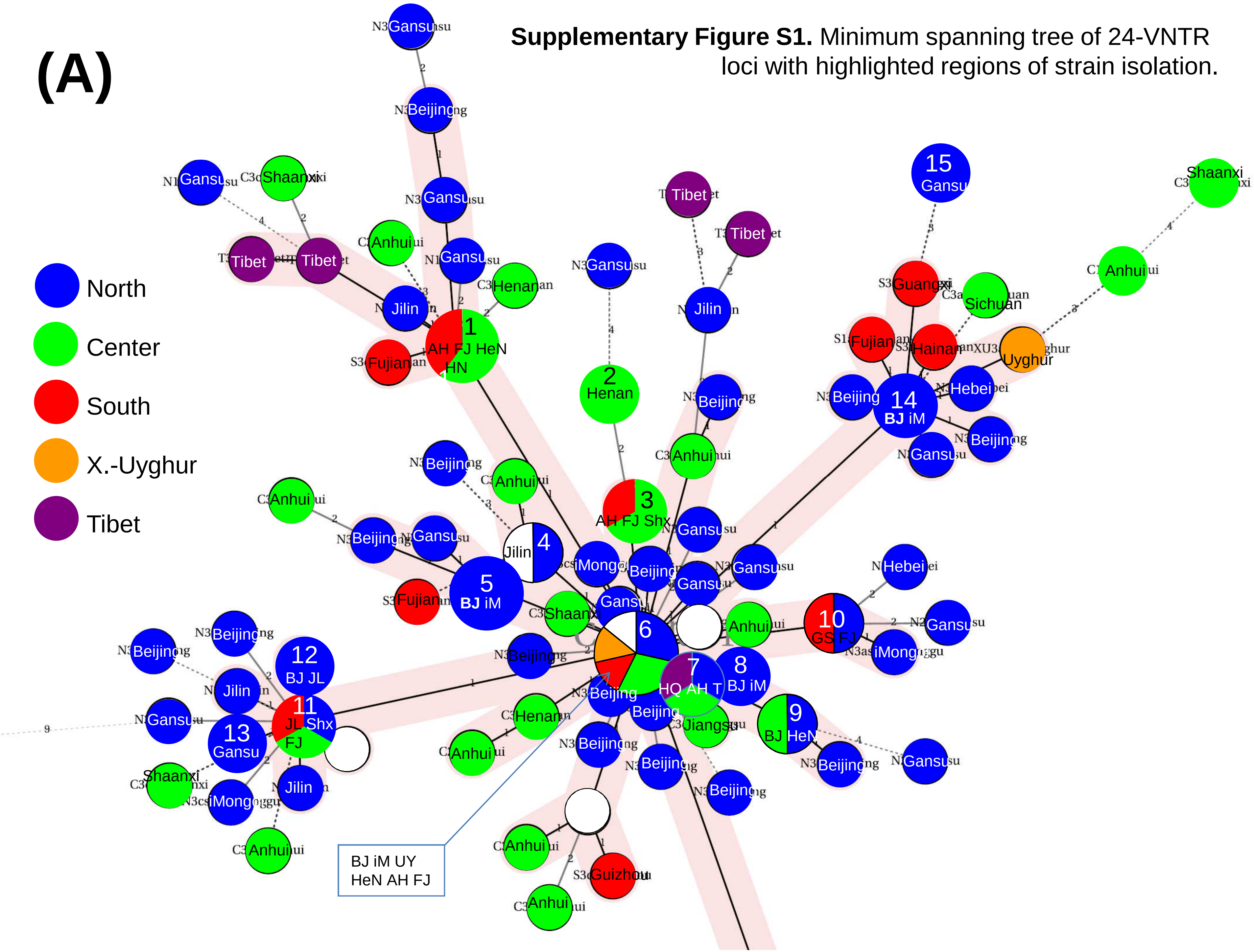

(B)

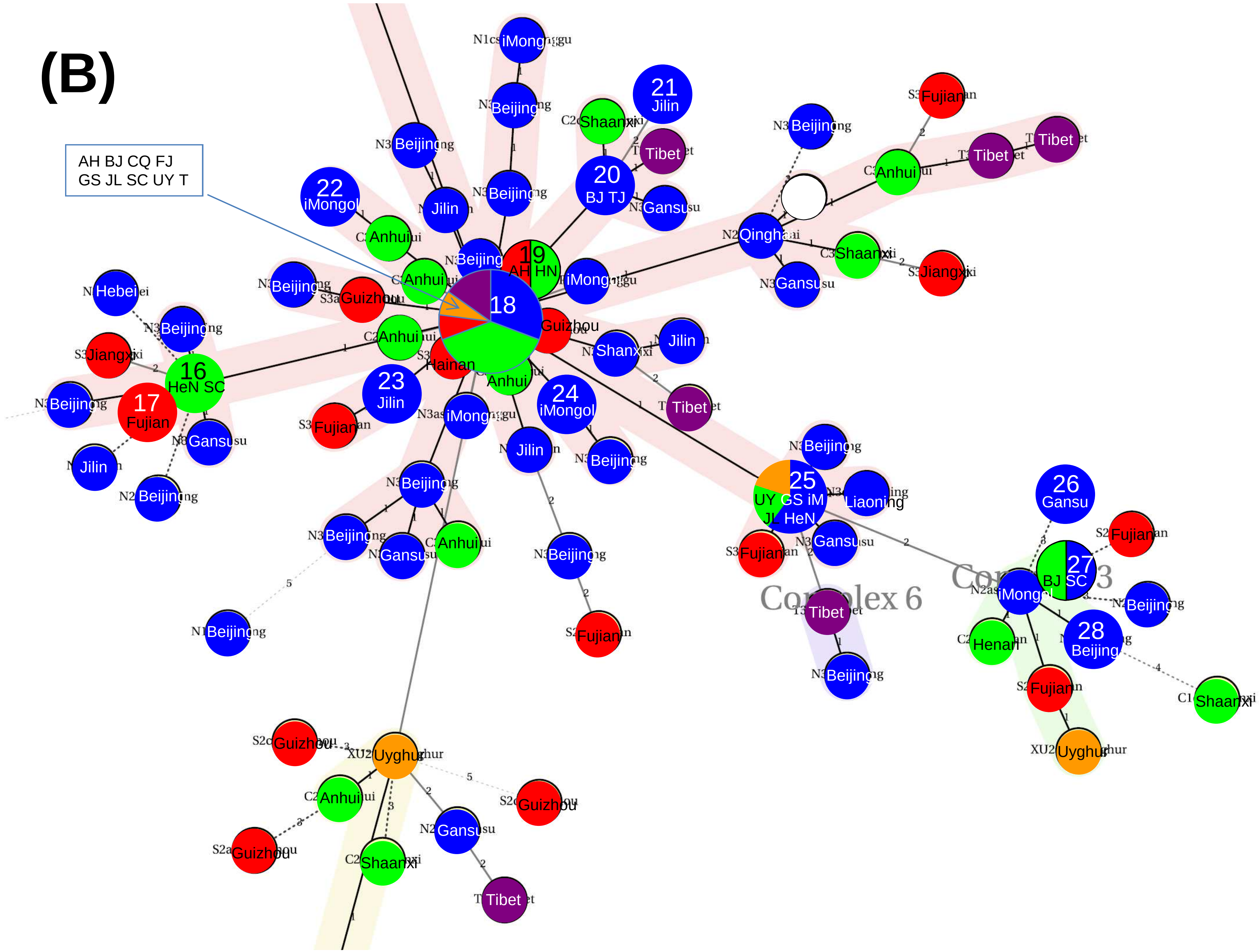

AH BJ CQ FJ
GS JL SC UY T
18
19 AH HN
20 BJ TJ
21 Jilin
22 iMongol
23 Jilin
24 iMongol
25 UY GS iM JL HeN
16 HeN SC
17 Fujian
26 Gansu
27 BJ SC
28 Beijing
Complex 6

(C)

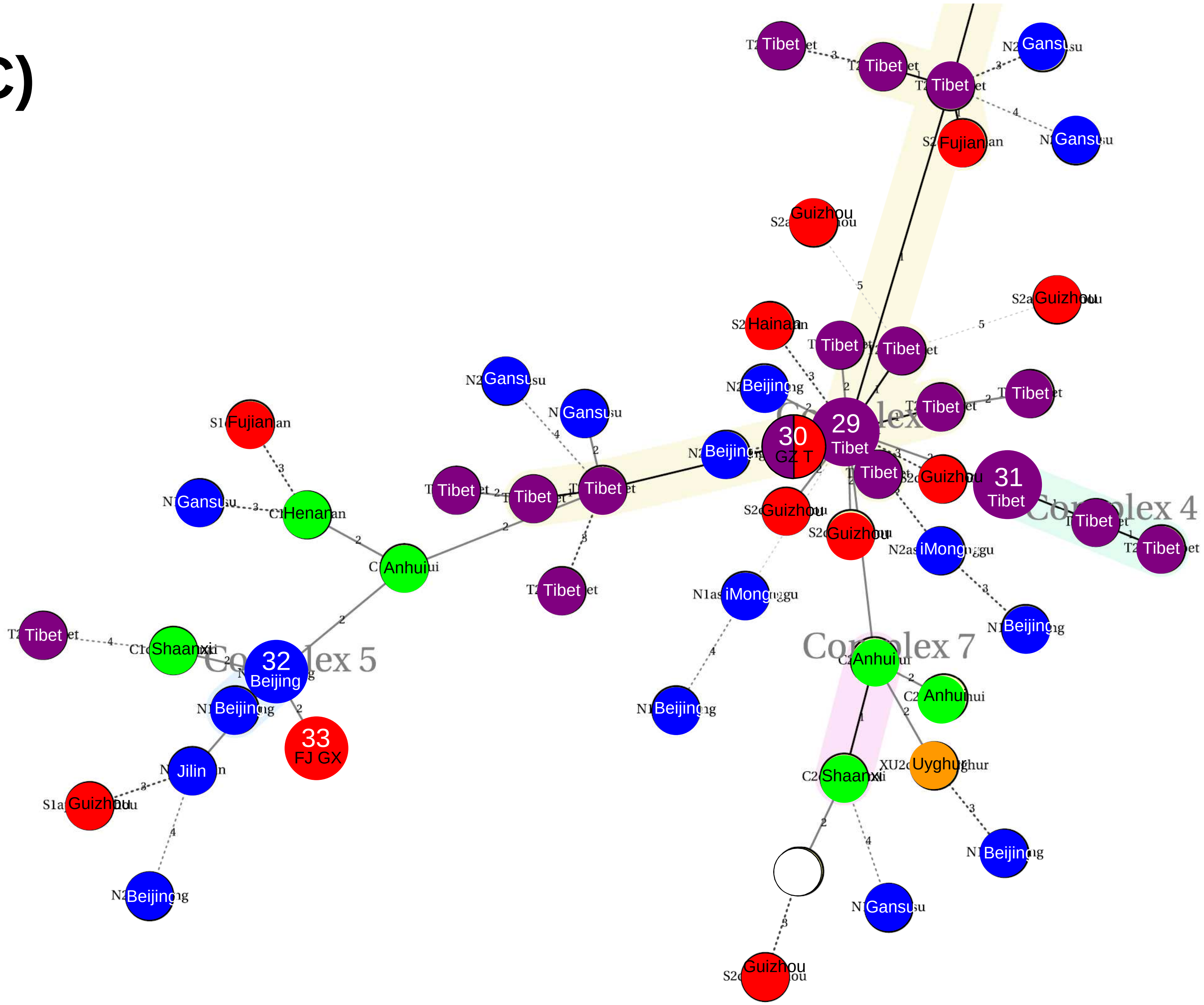

Complex 4
Complex 5
Complex 7
29
Tibet
30
GZ T
31
Tibet
32
Beijing
33
FJ GX
Tibet
Gansu
Fujian
Guizhou
Hainan
Beijing
Henan
Anhui
Shaanxi
Jilin
iMong
Uyghur

Supplementary Figure S2. Minimum spanning tree of 24-VNTR loci with highlighted Beijing genotype sublineages.

(A)

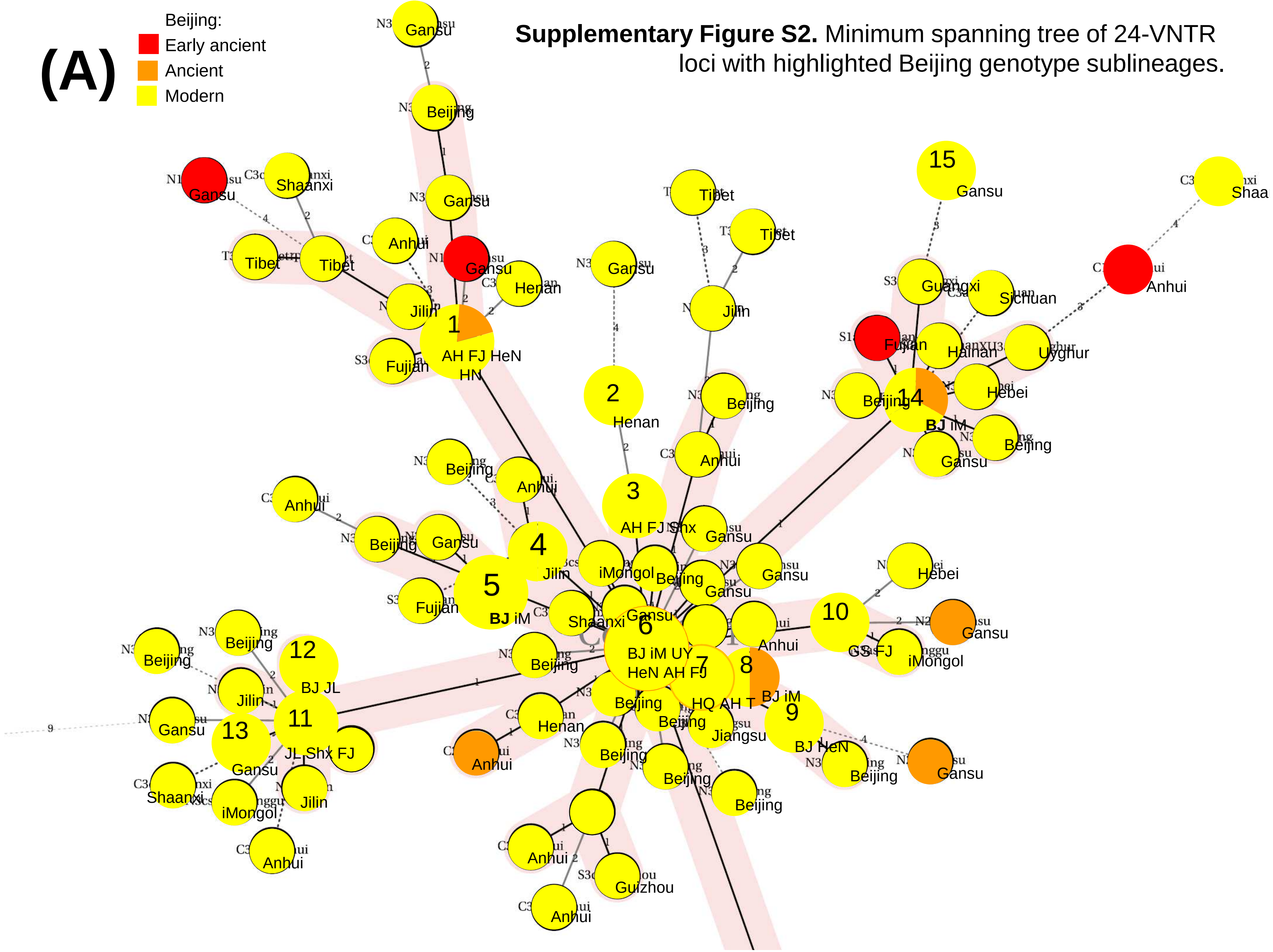

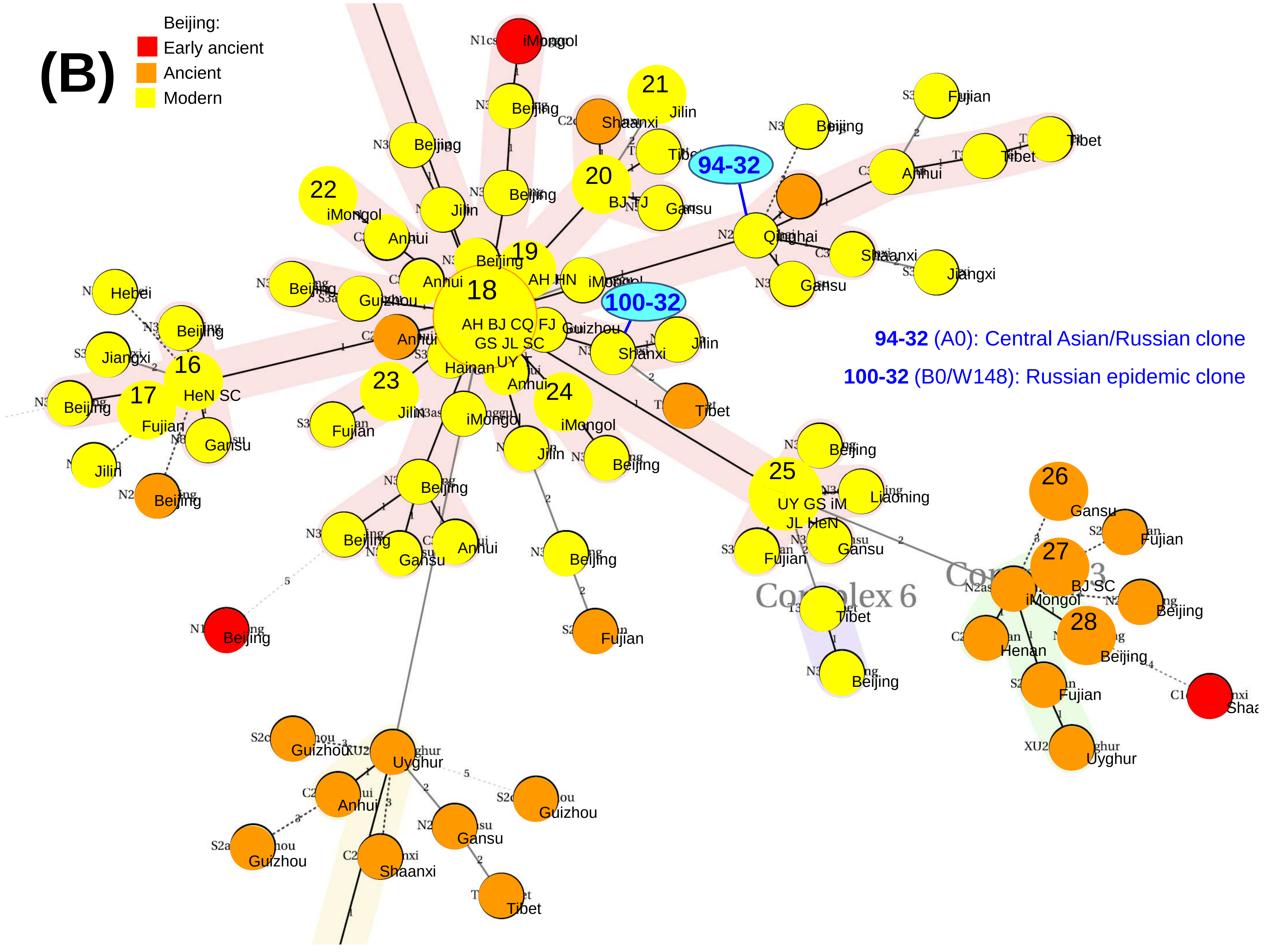
(B)
Beijing:
Early ancient
Ancient
Modern
94-32
100-32
94-32 (A0): Central Asian/Russian clone
100-32 (B0/W148): Russian epidemic clone
Complex 6
18
AH BJ CQ FJ
GS JL SC
UY
25
UY GS iM
JL HeN
16
HeN SC
17
Fujian
19
AH HN
20
BJ FJ
21
Jilin
22
iMongol
23
Jilin
24
iMongol
26
Gansu
27
BJ SC
28
Beijing

(C)

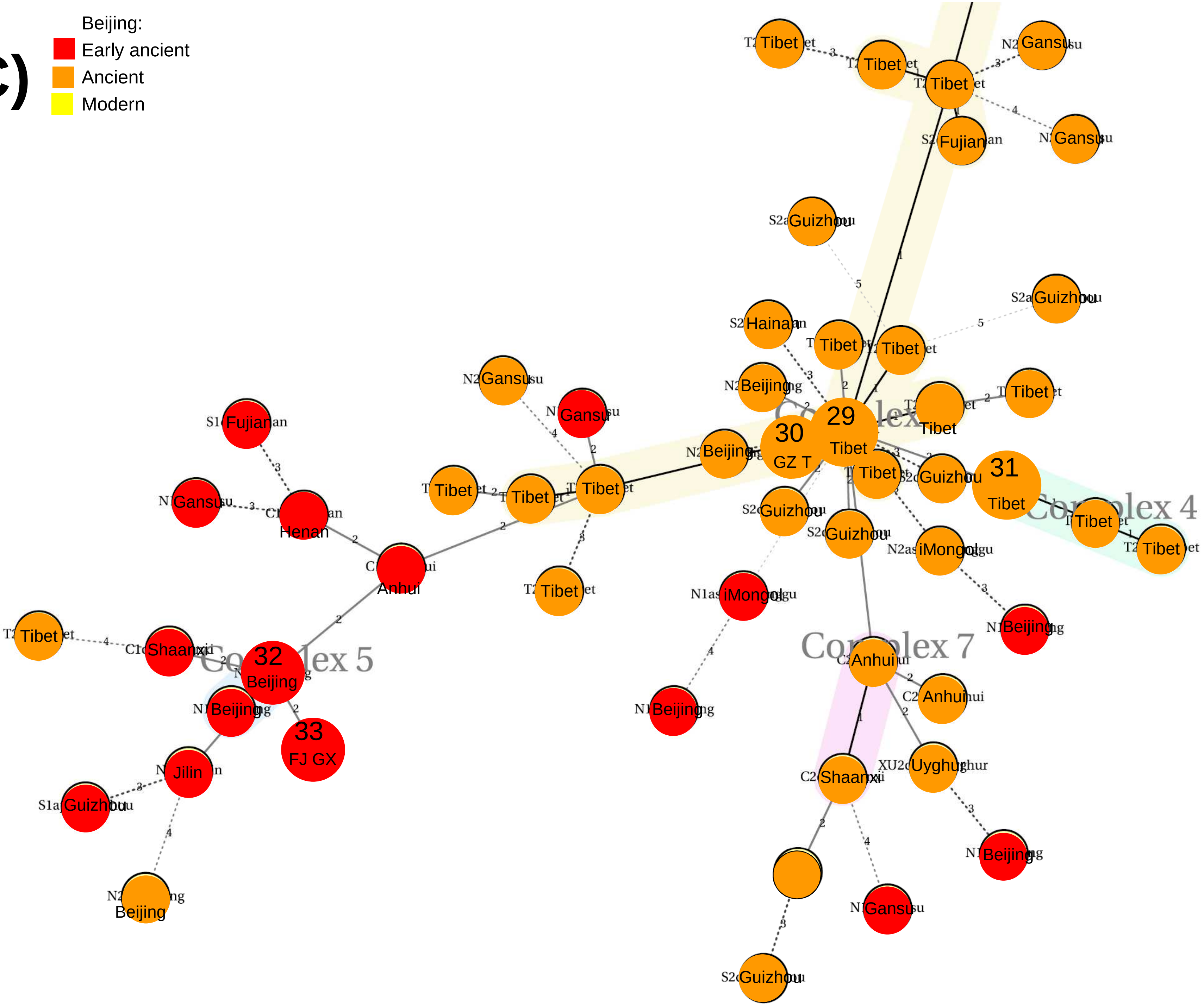
Beijing:
Early ancient
Ancient
Modern
29
Tibet
30
GZ T
31
Tibet
32
Beijing
33
FJ GX
Complex 4
Complex 5
Complex 7
Tibet
Gansu
Fujian
Guizhou
Hainan
Beijing
Henan
Anhui
Shaanxi
Jilin
iMongol
Uyghur

Supplementary Figure S3. Minimum spanning tree of 24-VNTR loci with highlighted age group (adult/child).

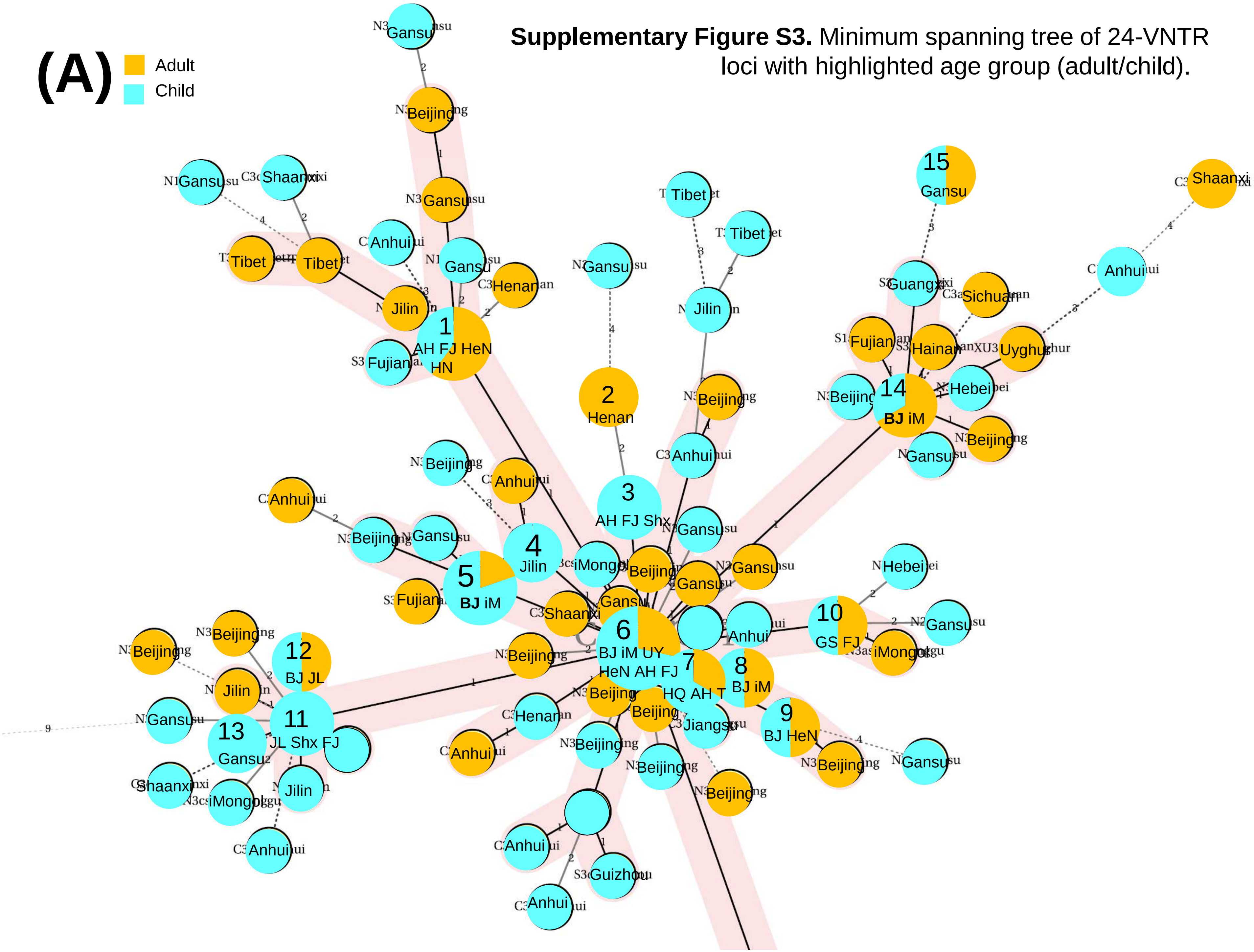

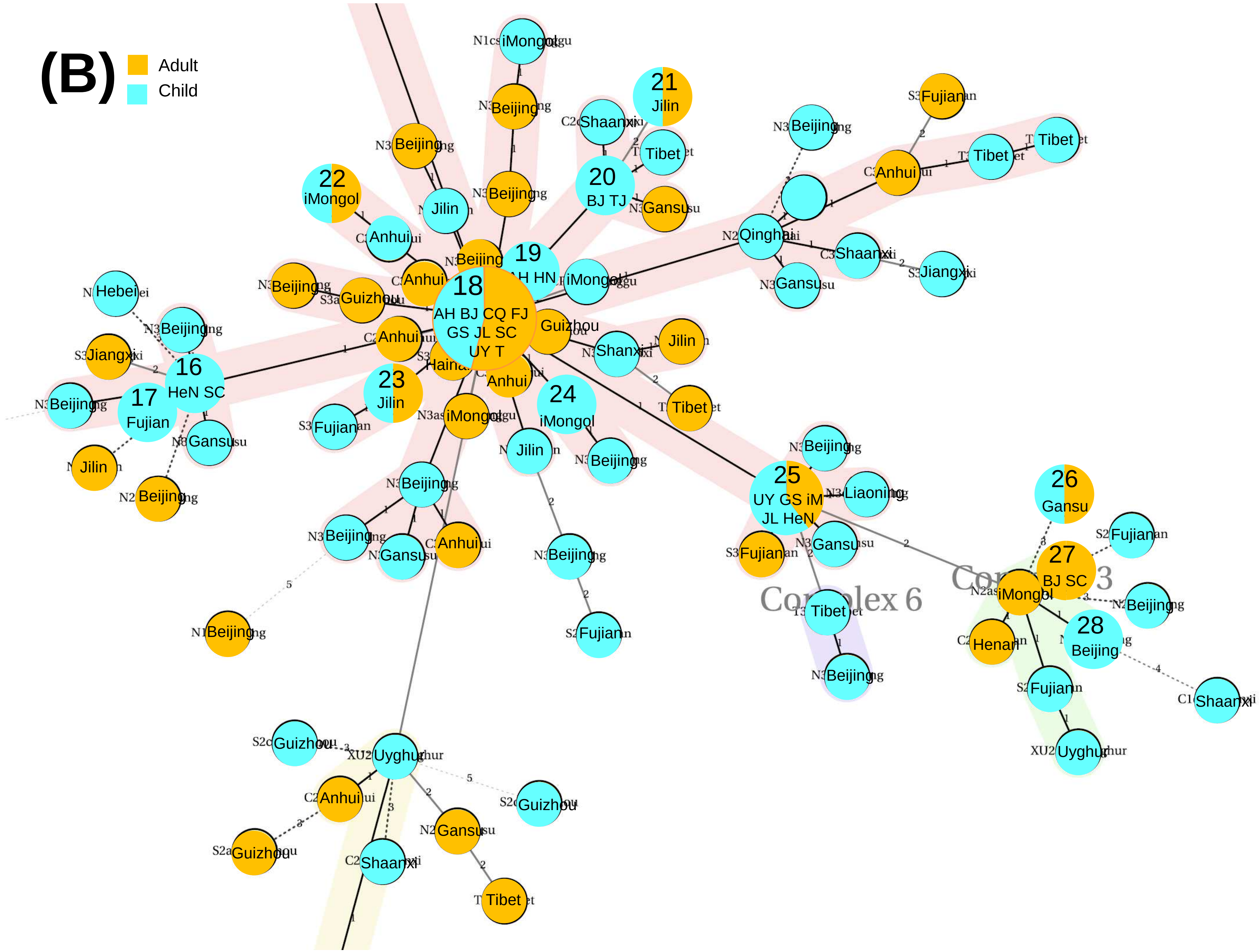
(B)
Adult
Child
16
HeN SC
17
Fujian
18
AH BJ CQ FJ
GS JL SC
UY T
19
20
BJ TJ
21
Jilin
22
iMongol
23
Jilin
24
iMongol
25
UY GS iM
JL HeN
26
Gansu
27
BJ SC
28
Beijing
Complex 6
Beijing
Jilin
Anhui
Guizhou
Gansu
Tibet
Fujian
Shaanxi
Jiangxi
Qinghai
Hebei
Liaoning
Henan
Uyghur
Shanxi

(C)

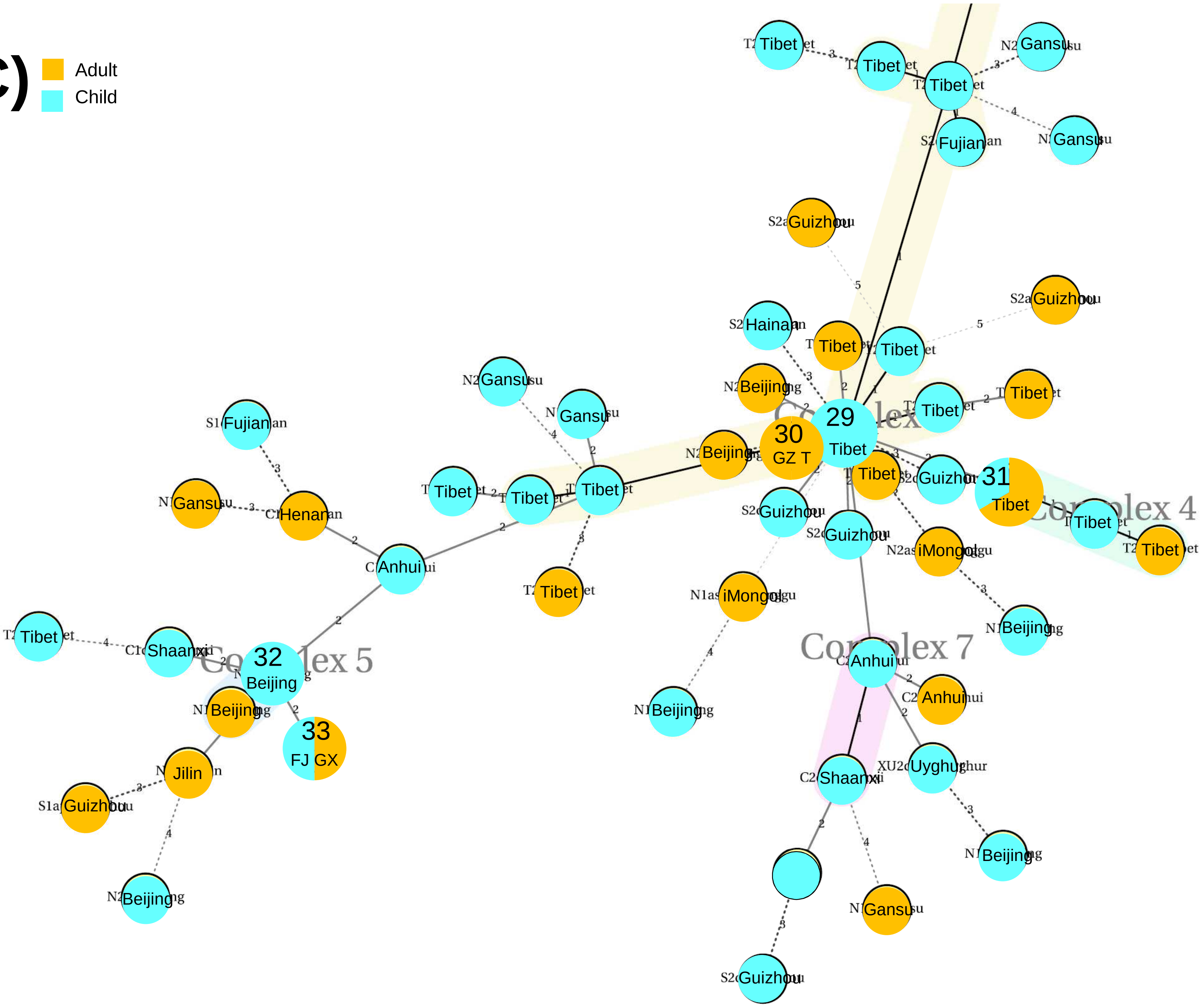

Adult
Child
29
Tibet
30
GZ T
31
Tibet
32
Beijing
33
FJ GX
Complex 4
Complex 5
Complex 7
Tibet
Gansu
Fujian
Guizhou
Hainan
Beijing
Henan
Anhui
Shaanxi
Jilin
iMongol
Uyghur

Supplementary Figure S4. Minimum spanning tree of 24-VNTR loci with highlighted drug resistance profile.

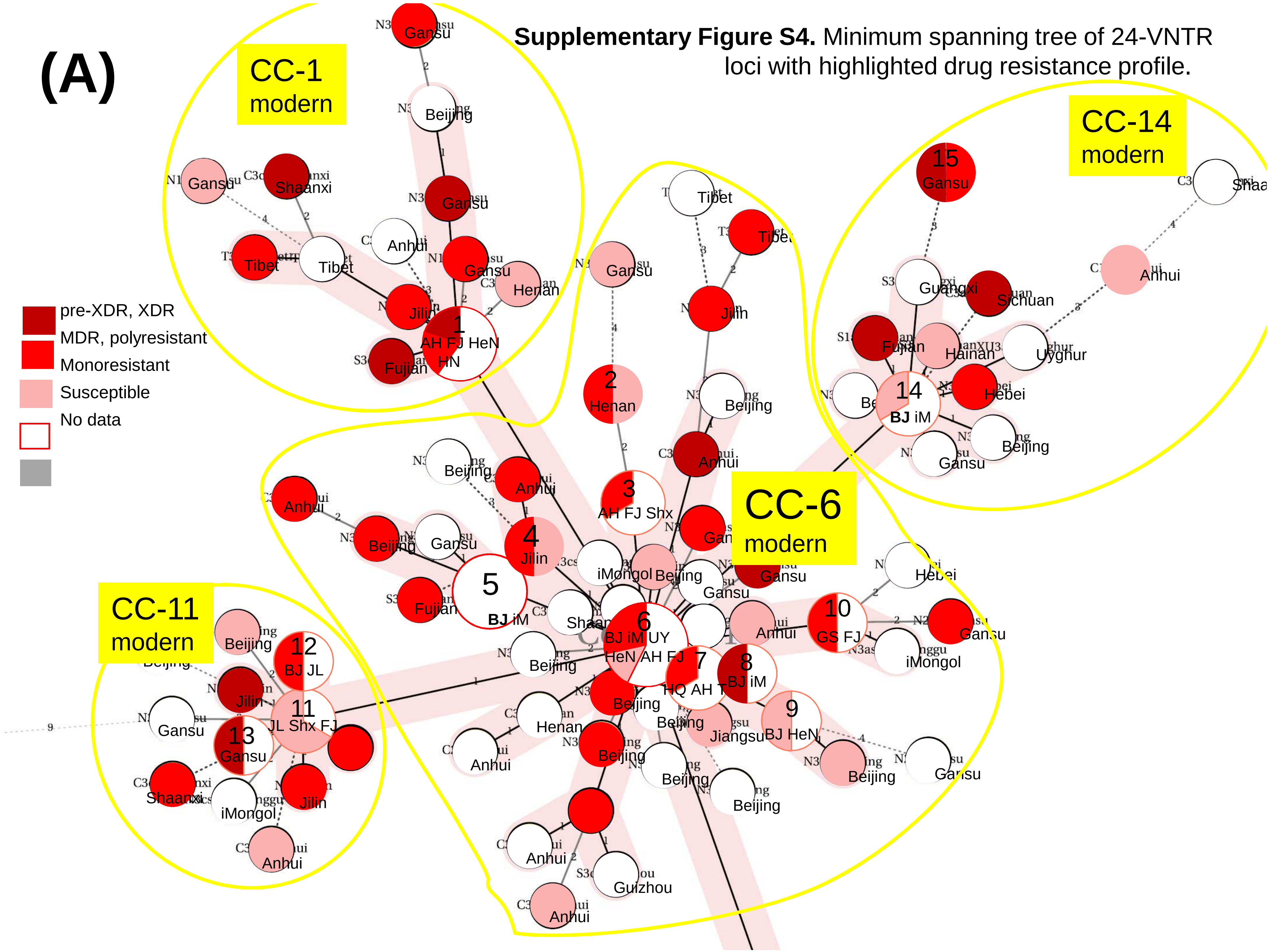

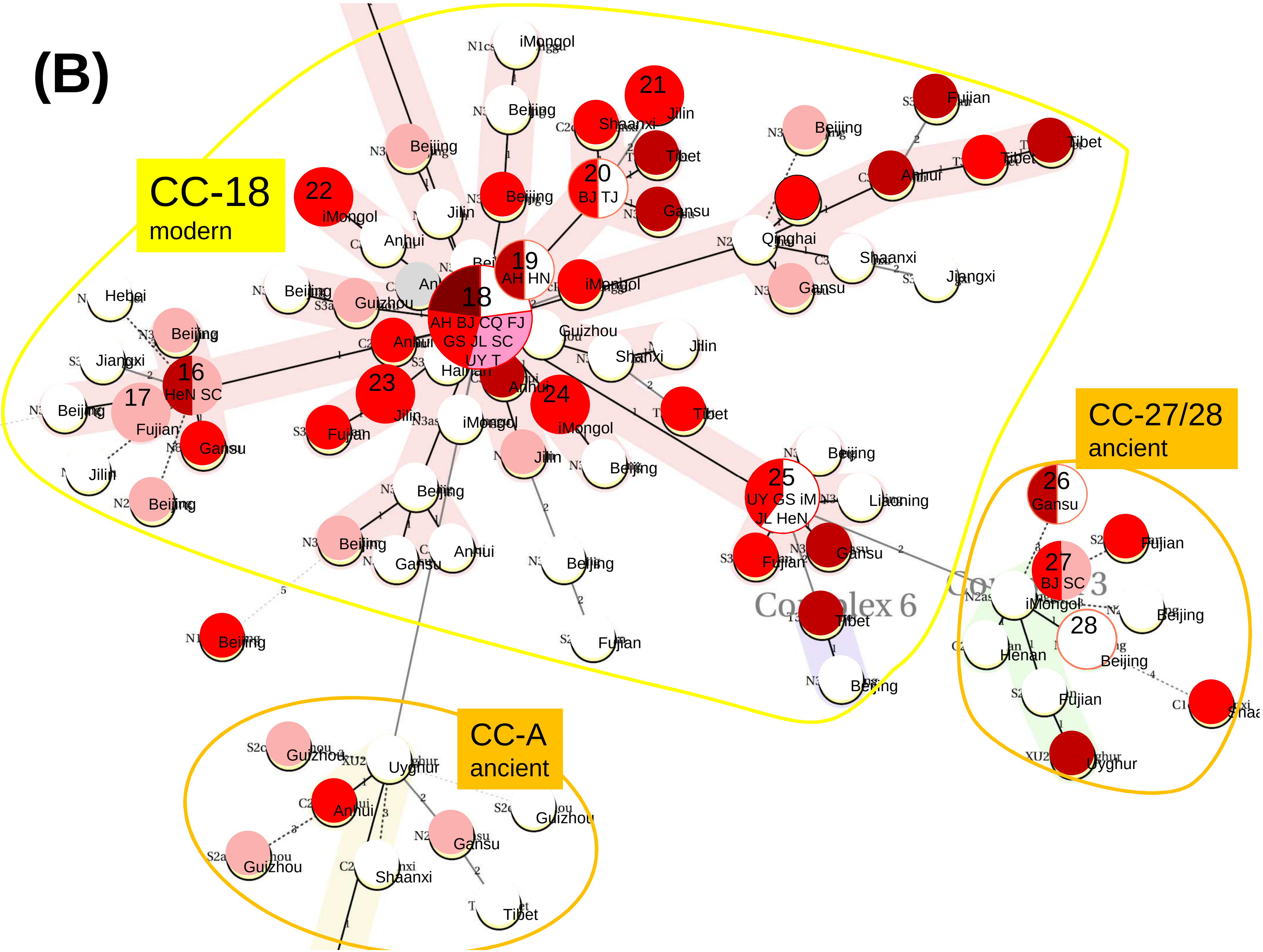

(B)
CC-18
modern
CC-27/28
ancient
CC-A
ancient
Complex 6
18
AH BJ CQ FJ
GS JL SC
UY T
19
AH HN
20
BJ TJ
21
22
23
24
25
UY GS iM
JL HeN
26
Gansu
27
BJ SC
28
16
HeN SC
17
Fujian
iMongol
Beijing
Jilin
Anhui
Shaanxi
Tibet
Gansu
Fujian
Qinghai
Jiangxi
Guizhou
Hainan
Hebei
Liaoning
Henan
Uyghur

(C)

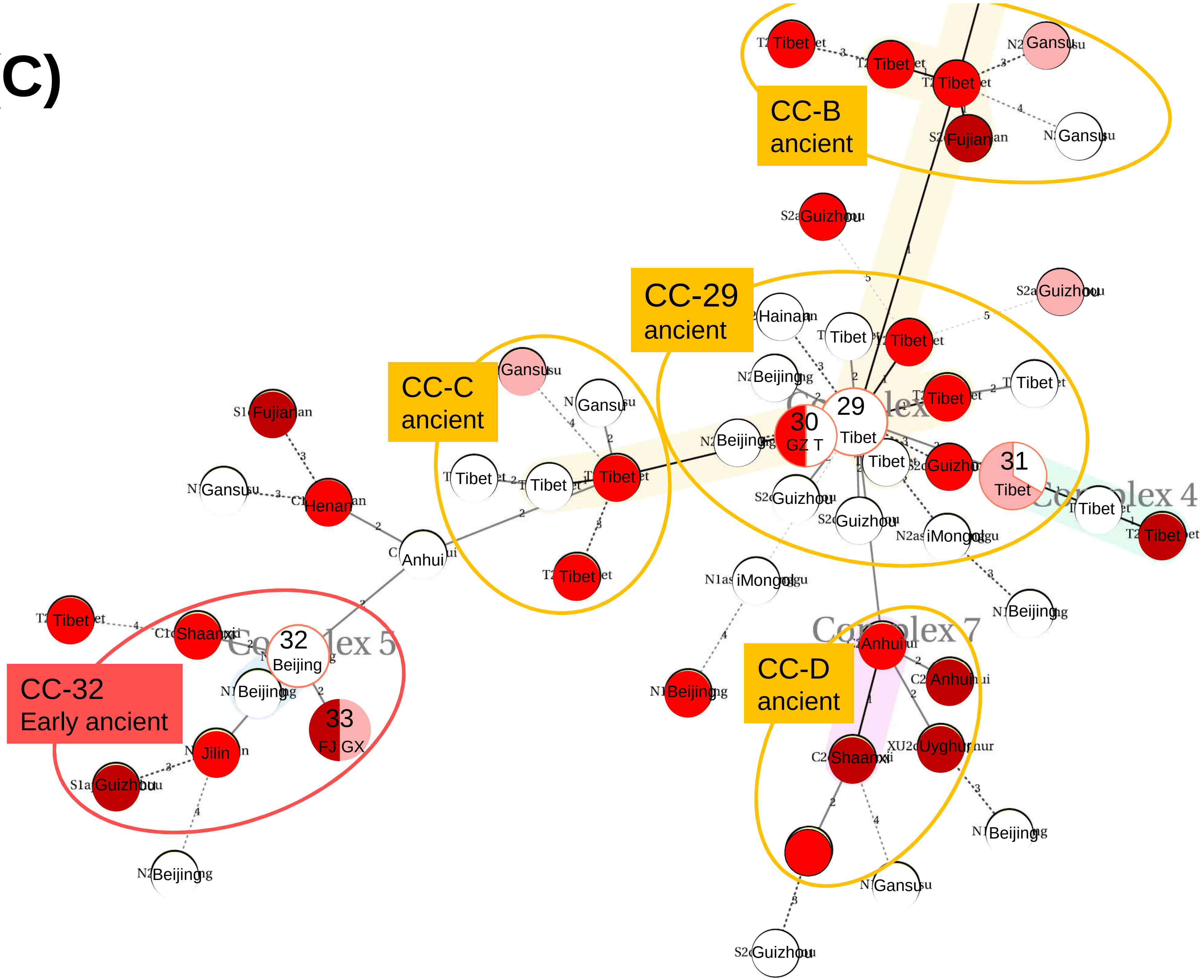
CC-B ancient
CC-29 ancient
CC-C ancient
CC-D ancient
CC-32 Early ancient
29 Tibet
30 GZ T
31 Tibet
32 Beijing
33 FJ GX
Tibet
Gansu
Fujian
Guizhou
Hainan
Beijing
Henan
Anhui
Shaanxi
Jilin
iMongol
Uyghur

Supplementary Figure S5. UPGMA dendrogram of 24-MIRU-VNTR profiles of M. tuberculosis Beijing genotype isolates from adult TB patients in China.
Number code designates Beijing sublineages: 1 - early ancient, 2 - ancient, 3 - modern. Mlva15-9 code was assigned by MIRU-VNTRplus.org. 24-loci order according to their order in genome.

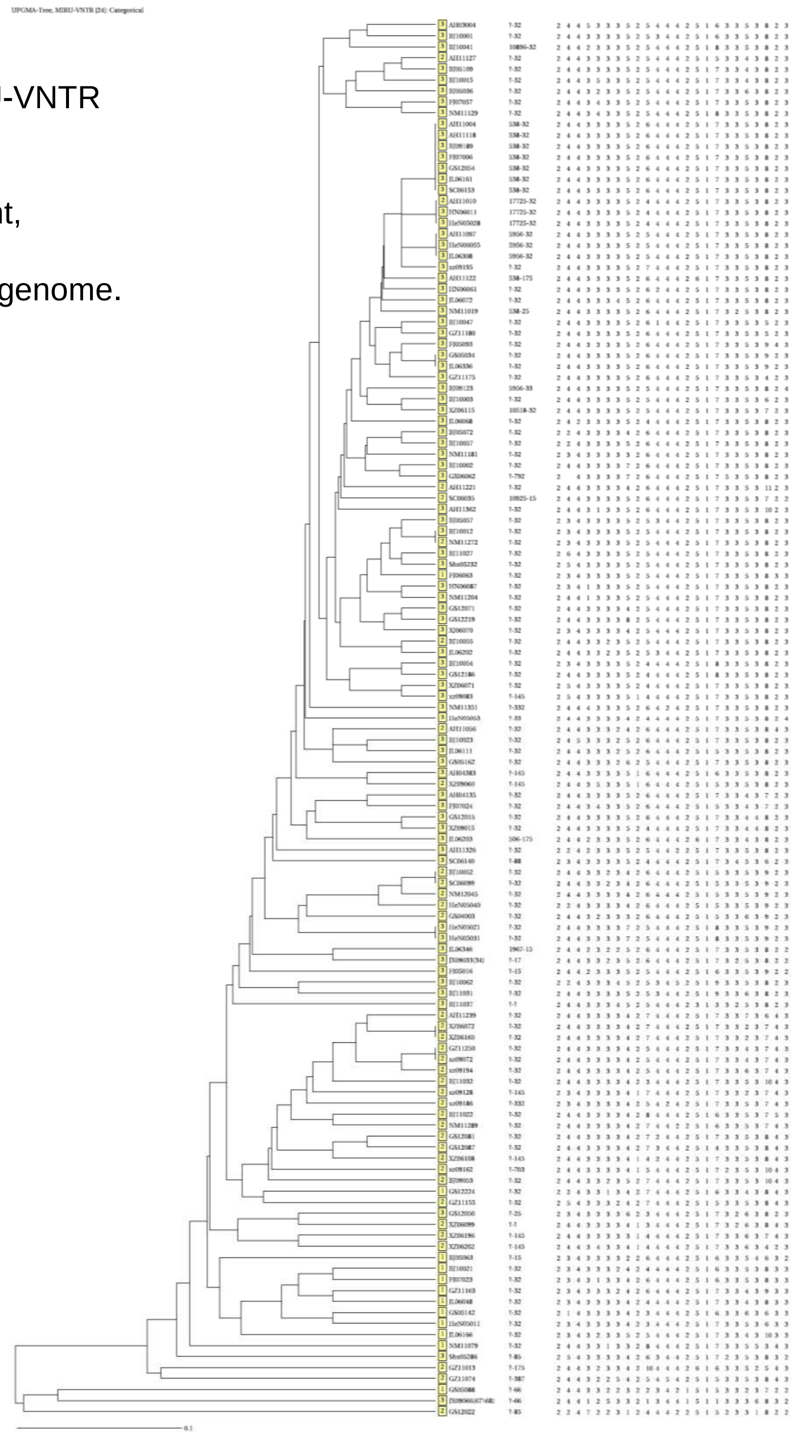

Supplementary Figure S6. UPGMA dendrogram of 24-MIRU-VNTR profiles of M. tuberculosis Beijing genotype isolates from children TB patients in China.
Number code designates Beijing sublineages: 1 - early ancient, 2 - ancient, 3 - modern. Mlva15-9 code was assigned by MIRU-VNTRplus.org. 24-loci order according to their order in genome.

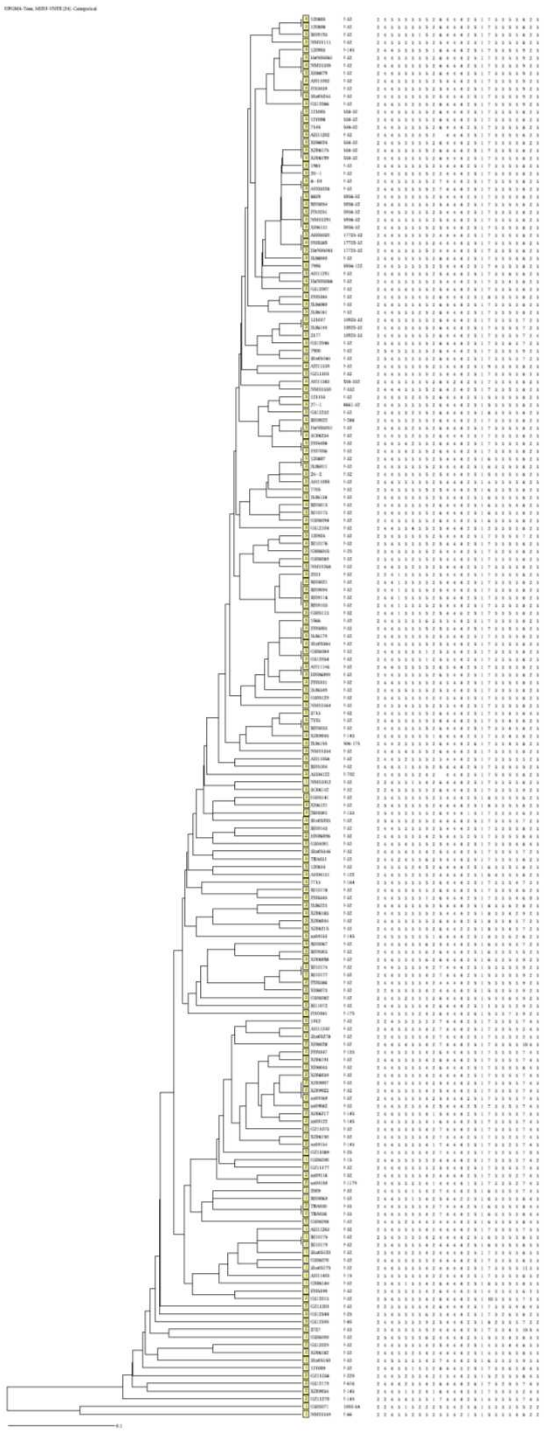

Supplement: Supplementary Table S2, Figures S1-S6 [file srep34353-s2.pdf]
